# Financing renewable energy: policy insights from Brazil and Nigeria

**DOI:** 10.1186/s13705-022-00379-9

**Published:** 2023-01-26

**Authors:** Abdulrasheed Isah, Michael O. Dioha, Ramit Debnath, Magnus C. Abraham-Dukuma, Hemen Mark Butu

**Affiliations:** 1 Energy and Technology Policy Group, ETH Zurich, Zurich, Switzerland; 2 Department of Economics, SOAS University of London, London, UK; 3 Department of Global Ecology, Carnegie Institution for Science, Stanford, CA USA; 4 Cambridge Zero, University of Cambridge, Cambridge, UK; 5 Energy Policy Research Group, Judge Business School, University of Cambridge, Cambridge, UK; 6 Department of Humanities and Social Sciences, California Institute of Technology, Pasadena, CA USA; 7 Just Transition Network, Jakarta, Indonesia; 8 Institute for Global Climate Change and Energy, Kyungpook National University, Daegu, Republic of Korea; 9 Africa Policy Research Institute, Berlin, Germany

**Keywords:** Renewable energy finance, Energy policy, Energy transition, Brazil, Nigeria

## Abstract

**Background:**

Achieving climate targets will require a rapid transition to clean energy. However, renewable energy (RE) firms face financial, policy, and economic barriers to mobilizing sufficient investment in low-carbon technologies, especially in low- and middle-income countries. Here, we analyze the challenges and successes of financing the energy transition in Nigeria and Brazil using three empirically grounded levers: financing environments, channels, and instruments.

**Results:**

While Brazil has leveraged innovative policy instruments to mobilize large-scale investment in RE, policy uncertainty and weak financing mechanisms have hindered RE investments in Nigeria. Specifically, Brazil’s energy transition has been driven by catalytic finance from the Brazilian Development Bank (BNDES). In contrast, bilateral agencies and multilateral development banks (MDBs) have been the largest financiers of renewables in Nigeria. Policy instruments and public–private partnerships need to be redesigned to attract finance and scale market opportunities for RE project developers in Nigeria.

**Conclusions:**

We conclude that robust policy frameworks, a dynamic public bank, strategic deployment of blended finance, and diversification of financing instruments would be essential to accelerate RE investment in Nigeria. Considering the crucial role of donors and MDBs in Nigeria, we propose a multi-stakeholder model to consolidate climate finance and facilitate the country’s energy transition.

## Background

Climate change poses an existential threat to humanity. The Paris Agreement aims to limit global warming to below 2 °C compared to pre-industrial temperatures to avoid cascading climate consequences. Achieving this will require significant energy decarbonization [1–3]. For example, it is estimated that achieving net-zero emissions by 2050 will require $4 trillion in annual clean energy transition investments by 2030 [1]. At the moment, investment in renewable energy (RE) falls well short of the scale needed to meet global climate targets [4]. This is due not only to the pace and scale of financing required, but also to path dependencies, short-termism, and risk aversion among policymakers and financial stakeholders [5]. However, underivestment in clean technologies is even more pronounced in low- and middle-income countries (LMICs) although the trajectory of their energy systems will have a significant impact on future emissions [1].

Public finance is fundamental to achieving the current energy transition. For example, public banks provide capital to project developers, mitigate investment risks, foster financial learning, and build confidence in low-carbon technologies [6–8]. Various types of public investment vehicles have accelerated technological change in solar and wind energy in recent decades [9]. Research also shows that direct public financing of renewables attracts disproportionate private investment [10]. Moreover, policymakers in developed economies are increasingly aligning public finance with climate goals [11].

On the other hand, project developers in LMICs still face daunting challenges in mobilizing investment in RE due to illiquid financial markets, technological risks, regulatory barriers, and volatile currencies [12, 13]. However, booming populations and rising energy demand present unique challenges for achieving universal electricity access and the Paris goals in Africa [14]. From an environmental Kuznets curve perspective, meeting rising energy demand with clean energy in LMICs could play a substantial role in decoupling future growth from high greenhouse gas emissions [15]. Resolving this dilemma requires research to inform the design of energy transition policies for LMICs. Nevertheless, knowledge of the risks, drivers, and effectiveness of policy mechanisms for accelerating financial flows to RE in LMICs is limited, as policy research on transition finance has mainly focused on Europe and North America [16]. Understanding the unique risk profiles, policy mixes, and stakeholder perspectives around clean technologies in these countries could inform policymaking to achieve energy and climate targets.

This paper compares the RE financing landscapes in Brazil and Nigeria. Comparing the two countries is important for three reasons. First, Nigeria and Brazil are the largest economies and most populous countries in Africa and South America, respectively, making their energy transitions relevant to global climate objectives. Second, both countries possess substantial RE resources, but have contrasting successes in attracting relative investment in clean technologies [17, 18]. Third, both countries have committed to increasing the share of renewables in their energy mix as part of their of Nationally Determined Contributions [19, 20].

We tackle three research questions: (1) What is the landscape of RE financing in Nigeria and Brazil? (2) What policies and support mechanisms have been used to deploy RE in Nigeria and Brazil, and what ensuing benefits and challenges have been observed for RE project developers? (3) What key policy lessons can Nigeria derive from Brazil? We contribute to the literature on finance and energy transition by providing actionable policy-relevant solutions to the challenges of attracting investments to accelerate the energy transition in LMICs. In addition, we propose a multi-pronged framework that fosters coordination among public and private actors, reduces transaction costs, and maximizes the efficient flows of public and private finance for RE project developers in Nigeria.

Following this introduction, “Methods” section presents an overview of the study’s framework. “Renewable energy in Nigeria and Brazil” section discusses the status of RE deployment and investment contexts in Brazil and Nigeria. “Results” section provides a detailed account of the RE financing environment, channels, and instruments employing our analytical framework. “Discussions” section outlines policy implications for Nigeria, while “Conclusion” section concludes the paper and provides an outlook for RE financing in Nigeria.

## Methods

### Study approach

**Fig. 1:**
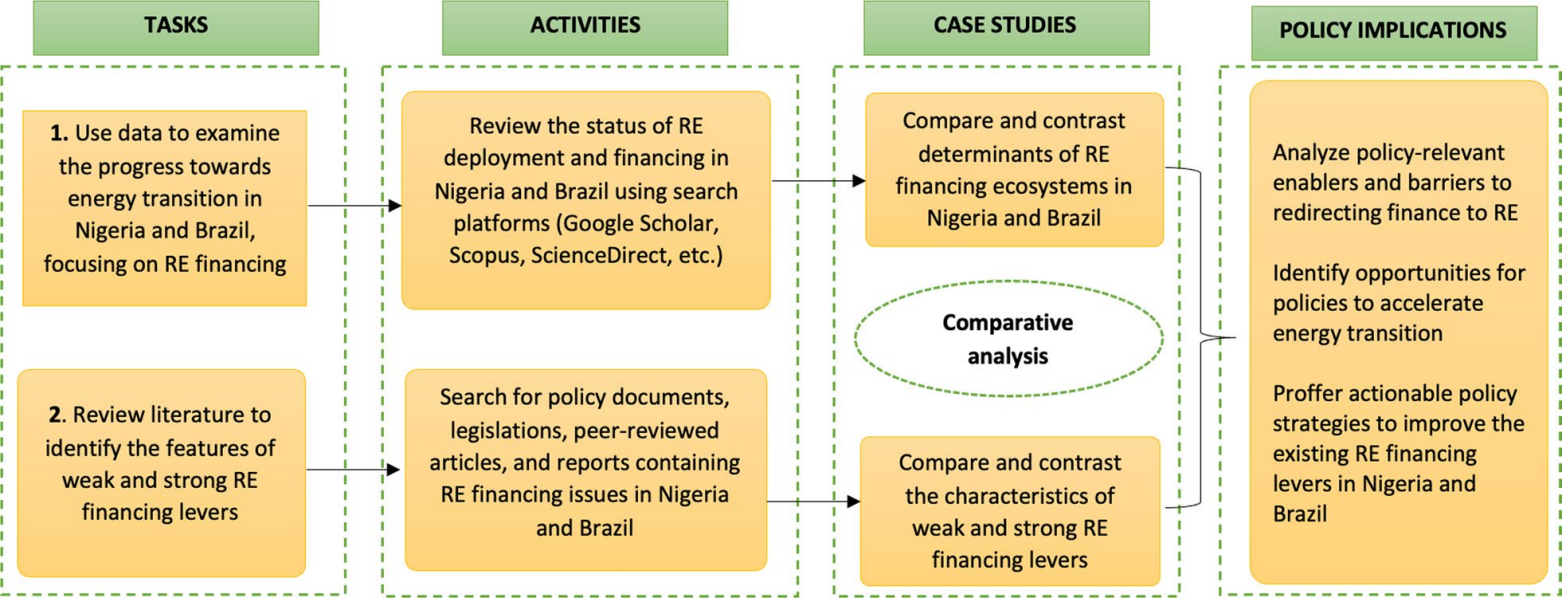
Overview of the study approach

This study employed a mixed-methods research design consisting of two main tasks (see Fig. 1). First, we examined the potential for energy transition in Nigeria and Brazil by reviewing the extent of renewables in the electricity matrix of both countries from 2000 to 2019. Second, we analyzed the evolution of energy policies in both countries, focusing on how institutional frameworks and policy tools have historically facilitated or hindered the deployment of low-carbon energy technologies. Consequently, we used a comprehensive analytical framework to evaluate the financing levers shaping RE projects, focusing on what Nigeria can learn from Brazil. We accomplished this through unstructured interviews, quantitative data analysis, and an extensive review of academic literature, policy documents, and energy regulations in Brazil and Nigeria.

We interviewed 12 relevant experts drawn from different sectors such as government, finance, think tanks, and civil society to ensure diversity of perspectives and avoid bias (see Table 1). Each interview lasted approximately one hour long, ranging from 40 to 60 minutes While our interviews were conducted between June 2018 and July 2020, we restricted our interview questions and discussions to the period 2000–2019 to factor out the effects of the pandemic on the energy transition. Five respondents were interviewed in person in London and Abuja, while seven were interviewed via video conferencing. To ensure anonymity, we coded respondents using the letter R with an integer based on their index among all respondents. For example, the first respondent was coded as R1, the second as R2, and so on. In addition, valuable information was obtained through several webinars and in-person workshops with policymakers, academics, and investors in London, Oxford, and Abuja. During the interviews, we centered our discussions around the three financing levers of our analytical framework, but other relevant policy issues affecting RE investment flows in both countries were also discussed. For example, regulatory frameworks around fuel subsidy reforms, local content, and green jobs were discussed. This approach to eliciting qualitative information enables researchers to collect comprehensive and high quality information from interviewees in a semi-structured format while minimizing leading question bias in the process [6].

**Table 1:** Interview respondents

No	Organization	Role	Date
1	Renewable energy enterprise	Chief Executive Officer	Nov. 2019
2	International development organization	Climate and Energy Adviser	Aug. 2018
3	Clean energy technical advisory	Country Manager	Apr. 2020
4	Energy and environment think tank	Research Fellow	Aug. 2018
5	Development bank	Clean Energy and Green Growth Expert	Jul. 2018
6	Rural electrification agency	Project Manager	May 2020
7	Investment bank	Head of Energy and Infrastructure	Apr. 2020
8	Renewable energy enterprise	Chief Operating Officer	Jun. 2018
9	Energy and environment think tank	Senior Research Fellow	Sep. 2019
10	Ministry of Finance	Energy Adviser	Dec.2018
11	Renewable energy association	President	Dec. 2018
12	Embassy	Economic and Trade Diplomat	Jan. 2019

Furthermore, we mapped the evolution of RE policies in Nigeria and Brazil using official publications and repositories of government authorities. This was undertaken in two stages.

First, we conducted an extensive search for key RE policies, laws, and regulatory frameworks to create a database to guide the empirical analysis of RE policy evolution in the two countries. For Nigeria, we searched the websites of the Nigerian Electricity Regulatory Commission (NERC), the Energy Commission of Nigeria (ECN), Ministry of Power (MoP), the Ministry of Environment (MoE), the Rural Electrification Agency (REA), and the Nigeria Bulk Electricity Trading (NBET). For Brazil, we searched the websites of the Brazilian Electricity Regulatory Agency (ANEEL), the Ministry of Mines and Energy (MME), and the Energy Research Office (EPE). We used keywords such as “renewable energy” combined with “policy”, “regulation”, “investment”, “masterplan”, and “incentives”. We triangulated our database by reviewing other peer-reviewed articles and official reports on energy policies in Brazil and Nigeria. Second, we synthesized the policies by identifying principal RE objectives and specific policy mechanisms, such as feed-in tariffs, tax incentives, and financing instruments in both countries. Finally, we explored existing scientific literature to ascertain policy parameters and enabling conditions for fostering the clean energy transition, specifically focusing on the three financing levers summarized in our analytical framework.

### Analytical framework

**Fig. 2:**
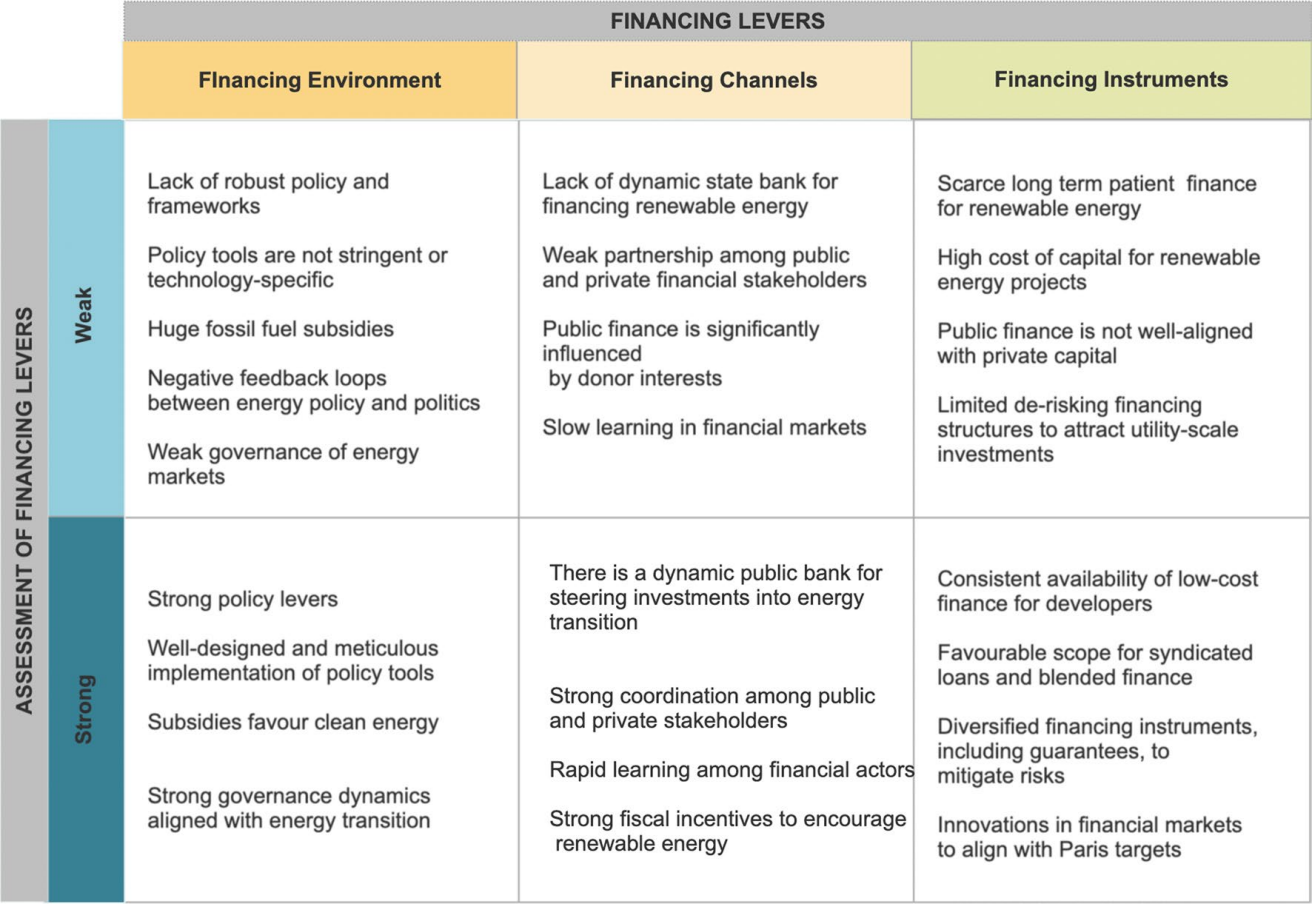
Analytical framework

We extend the typology developed by Liming [21] to examine energy transition and the policy environment. Our framework enables a holistic analysis of policy-relevant issues affecting energy transitions in LMICs based on three distinct but interrelated financing levers: financing environment, channels, and instruments (see Fig. 2). The three-part framework broadly covers policy and regulatory frameworks, market incentives, risk fundamentals, and public and private stakeholders that are relevant to constrain or enable energy transition [22, 23]. Drawing on existing literature, our framework categorizes each financing lever as either weak or strong depending on whether the relevant actors and parameters of interest enable or restrict investment in renewables. For clarity, we summarize the financing levers as follows:

#### Financing environment

This refers to the regulatory, governance, and policy frameworks that influence capital flows into energy transition-related infrastructure. Socio-technical transition theories conceptualize the energy transition as a dynamic outcome of multi-level interactions transcending established technological regimes and niches, incumbent fossil-based energy actors and socio-technical low-carbon innovators, operating under specific institutional frameworks [24]. Geddes and Schmidt [8] argue that finance is critical for niche-regime interactions. Specifically, the reallocation of finance toward niche low-carbon technologies requires either that the niche conforms to the financing regime or that the regime be redesigned to support niche technologies. Thus, renewables will scale rapidly if governance institutions implement robust policy tools that favor disruptive clean technologies over incumbent fossil fuels. In the current energy transition, policy tools such as subsidies and tax breaks have significantly contributed to the global deployment of renewables by de-risking the financing environment for project developers. A weak financing environment compounds technology risks, market barriers, and the risk-return profiles of RE investments, thus impeding financial flows into renewable technologies. On the other hand, a strong financing environment reduces investment risks and attracts private capital into renewables [25]. More broadly, strong financing environments make RE technologies competitive with fossil-fuel energy sources.

#### Financing channels

These constitute the ecosystems of actors that invest in RE technologies using specific instruments. Essentially, they include public, private, and community stakeholders with distinct but intertwined stakes in financing the clean energy transition. It is generally recognized that achieving the current transition will require financial institutions to redirect finance away from fossil fuels to renewables [4]. In weak financing channels, there is insufficient momentum among financial stakeholders to channel capital into green projects [13, 26]. This could be due to short-termism, slow learning, path dependencies, and insufficient awareness of low-carbon transition risks in the financial services industry [27, 28]. Similarly, poor coordination between public and private stakeholders is a major correlate of weak financing channels, especially in the absence of a dynamic public entity to deploy patient capital and galvanize private investment [6].

On the other hand, strong financing channels often have dynamic green state investment banks, robust public–private coordination, and rapid learning among financial stakeholders [8, 27]. In such contexts, there is a positive feedback mechanism between “green coalitions” stakeholders, regulatory frameworks, and policy-induced technological change in the energy sector [29]. The broad spectrum of financial stakeholders in the RE sector in LMICs includes multilateral development banks (MDBs), financial intermediaries, state-owned banks, private equity, and institutional investors [26].

#### Financing instruments

These are the public and private financial mechanisms for financing renewables or attracting additional private capital. Public instruments aim to tackle systemic investment risks associated with low-carbon technologies across the supply chain, from ideation to deployment [9]. For example, they could directly finance renewables R&D or shape market incentives by lowering the cost of capital to de-risk investment in low-carbon technologies. Typical examples include grants, subsidies, tax incentives, and guarantees [25]. Private instruments concern the corporate and project finance structures that RE firms use to raise equity and debt finance [30]. These include equity, retained earnings, loans, special purpose vehicles, bonds, and senior debts. We propose that diversification of financing instruments is crucial to mitigate the risks associated with RE projects in developing countries and to attract capital investment from a broad spectrum of investors. Essentially, countries with weak financing instruments tend to experience a shortage of catalytic finance, weak alignment of public and private financing arrangements, high cost of capital, and limited bankable projects to attract large-scale investments [8, 22]. For instance, in developing countries, RE investments are often low due to insufficient guarantees to hedge against structural risks, such as exchange rate volatilities and political instability [26]. On the other hand, countries with strong financing instruments often have abundant concessional capital, robust financing partnerships between public and private institutions and diversified financial mechanisms to mitigate risks for investors. For a detailed discussion of different financing instruments for RE technologies, we refer the reader to read [25, 30].

## Renewable energy in Nigeria and Brazil

**Fig. 3:**
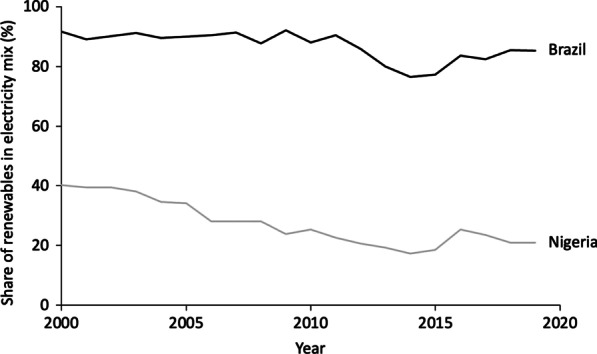
Share of renewables in electricity mix of Brazil and Nigeria (2000–2019)

Both Nigeria and Brazil have significant renewable electricity potential. While Brazil has made remarkable progress in increasing its share of renewables, especially wind and solar, Nigeria has yet to make meaningful headway. Nigeria’s electricity mix is dominated by gas-fired power plants and hydropower dams, contributing 81% and 19% of electricity generation, respectively, as of 2019 [18]. Nigeria’s abundant RE resources remain untapped, with solar and wind technologies contributing less than 1% of electricity generation (see Fig. 3). Unlocking investment in these RE sources would be significant in providing clean, safe, and affordable energy for Nigerians [31, 32].

Brazil’s energy sector has experienced significant transformations in recent decades. Thanks to the Light for All (*Luz Para Todos*) electrification program, the country achieved universal electricity access in 2014 through the rapid deployment of off-grid solar technologies in remote communities, especially in the Amazonian regions [33]. As a result, Brazil has one of the least carbon-intensive power sectors in the world [34]. While non-hydro renewables contribute a small share of its energy mix, they have been growing in the last decade due to favorable policies, technological innovation, and rising electricity demand [33].

## Results

In this section, we apply our analytical framework to analyze the how financing environment, channels, and instruments shape the evolution, type, and volume of clean energy investment in the two countries.

### Financing environment for renewable energy

#### Nigeria’s financing environment

**Fig. 4:**
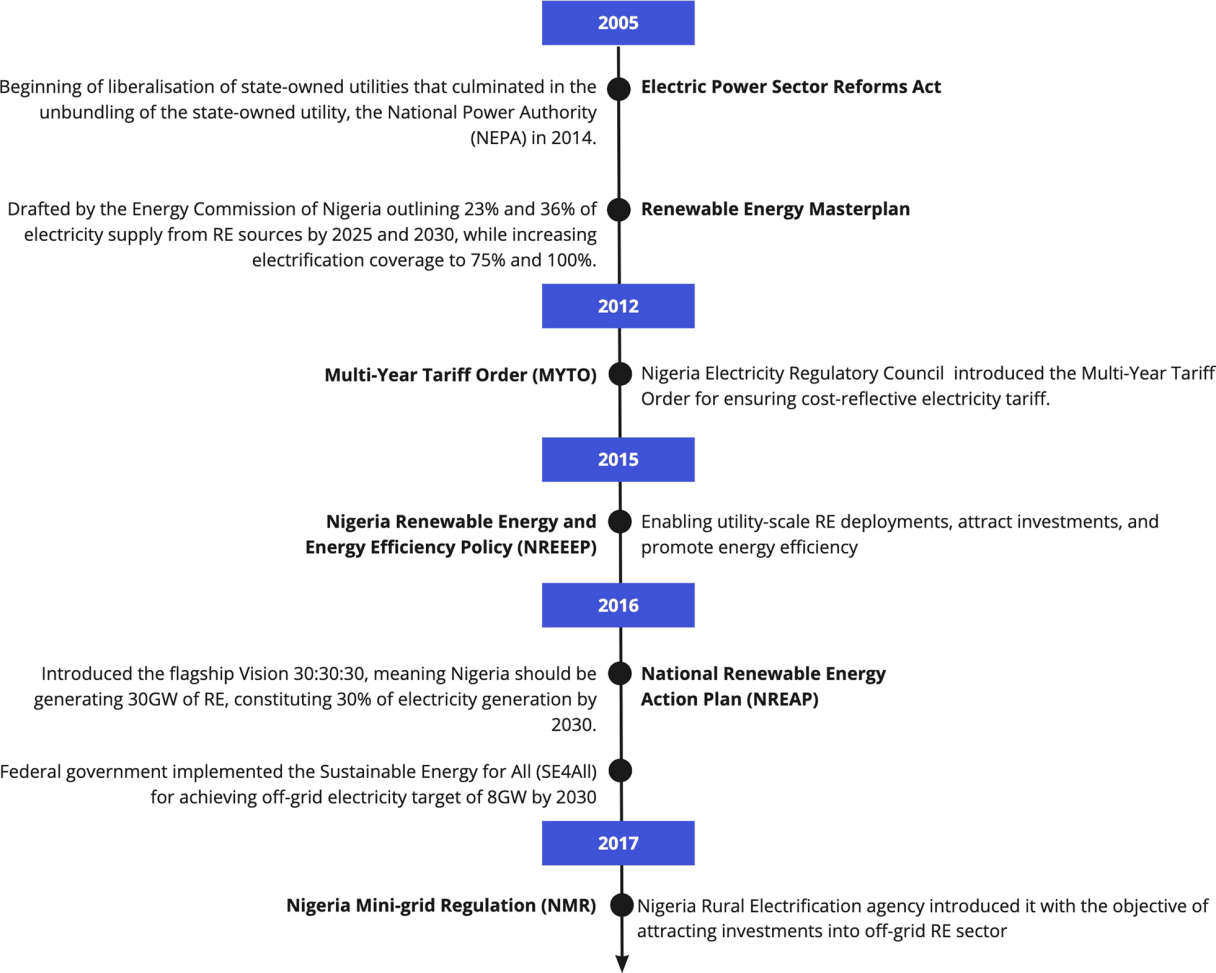
Key RE financing policy frameworks in Nigeria

Over the past two decades, Nigerian authorities have introduced several policies and regulations targeted at creating a conducive financing environment for renewables deployment. However, it is widely acknowledged that such efforts have achieved limited success [18, 35]. Figure 4 provides a timeline of key energy policies that shaped the RE financing environment in Nigeria.

Major policy reforms in the Nigerian electricity market began in 2005 with the promulgation of the Electric Power Sector Reform Act, which unbundled and privatized the National Electric Power Authority (NEPA). Since 2014, six Generation Companies (GenCos) and eleven Distribution Companies (DisCos) have controlled electricity generation and distribution. Although the electricity sector was privatized to improve market efficiency, there have been limited improvements in electricity access and service delivery [36]. This could be attributed to governance failures due to corruption and clientelism that hindered the transparent privatization of the electricity assets [37, 38]. R5 noted that *“electricity assets were cheaply sold off to politically-connected stakeholders with neither track record in electricity management nor genuine interests in long-term efficiency to improving services to end-users”*. As such, numerous constraining inefficiencies in the electricity market have increased investment risks for RE projects [36, 38]. For example, while the installed electricity generation capacity is about 12 GW, the GenCos barely generate 5 GW, and DisCos have been unable to raise sufficient revenues due to decrepit grid network, high transmission and distribution (T&D) losses, and mounting debt stress in the electricity industry [36]. Between 2015 and 2018, the power sector received emergency bailouts from the federal government amounting to around $3.9 billion [37].

Furthermore, the Renewable Energy Masterplan (RMP) of 2005 faced binding institutional bottlenecks. Specifically, R5 argued that the policy document faced bureaucratic delays in the parliament before it was approved seven years later. The RE targets in the RMP were missed due to insufficient political support and market incentives for investors [22, 32].

The Nigeria Electricity Regulatory Council (NERC) introduced the Multi-Year Tariff Order (MYTO) in June 2012 to attract investment into the electricity markets. According to interviewee 4, “*MYTO was principally designed to ensure cost-reflective tariffs and to attract investments into the electricity markets, including from clean energy sources, but lackluster implementation has always been its bane*.” Indeed, subsequent policies provided horizontal support mechanisms for renewables, such as FiTs, tax incentives, and net metering regulations [39]. It was argued that the policy objective was to attract RE deployment and promote energy efficiency in the country [40]. Relatedly, the Nigeria Renewable Energy and Energy Efficiency Policy (NREEP) pledged to overcome financing barriers for developers by providing concessional loans from the Power Sector Development Fund [39]. Nonetheless, details on financing incentives and the role of different stakeholders in achieving the RE targets were lacking. Arguably, the most promising institutional framework was the Regulation for Mini-Grids, which was specifically designed to attract investments into Nigeria’s off-grid RE sector by addressing challenges related to tariffs, grid connections, and environmental licensing [41]. Yet, it remains to be seen whether policymakers will be committed to implementing the regulatory frameworks and to what extent investment risks for off-grid RE enterprises will be mitigated (R4, R5, and R8).

In summary, the weak financing environment for renewables in Nigeria could be traced to poor RE policy design and implementation. This was echoed in our interviews with R5, R6, and R7, who specifically mentioned institutional bottlenecks, such as the protracted implementation of cost-reflective electricity tariffs, policy reversals related to the power purchase agreement (PPA) with eleven solar project developers, and misaligned tariffs for solar technologies. Indeed, [42] argues that governance has always influenced the adoption of energy technologies in Nigeria over the past decades. Other challenges in the financing environment include scarce private capital, exchange rate volatilities, and insecure land rights [43].

#### Brazil’s financing environment

**Fig. 5:**
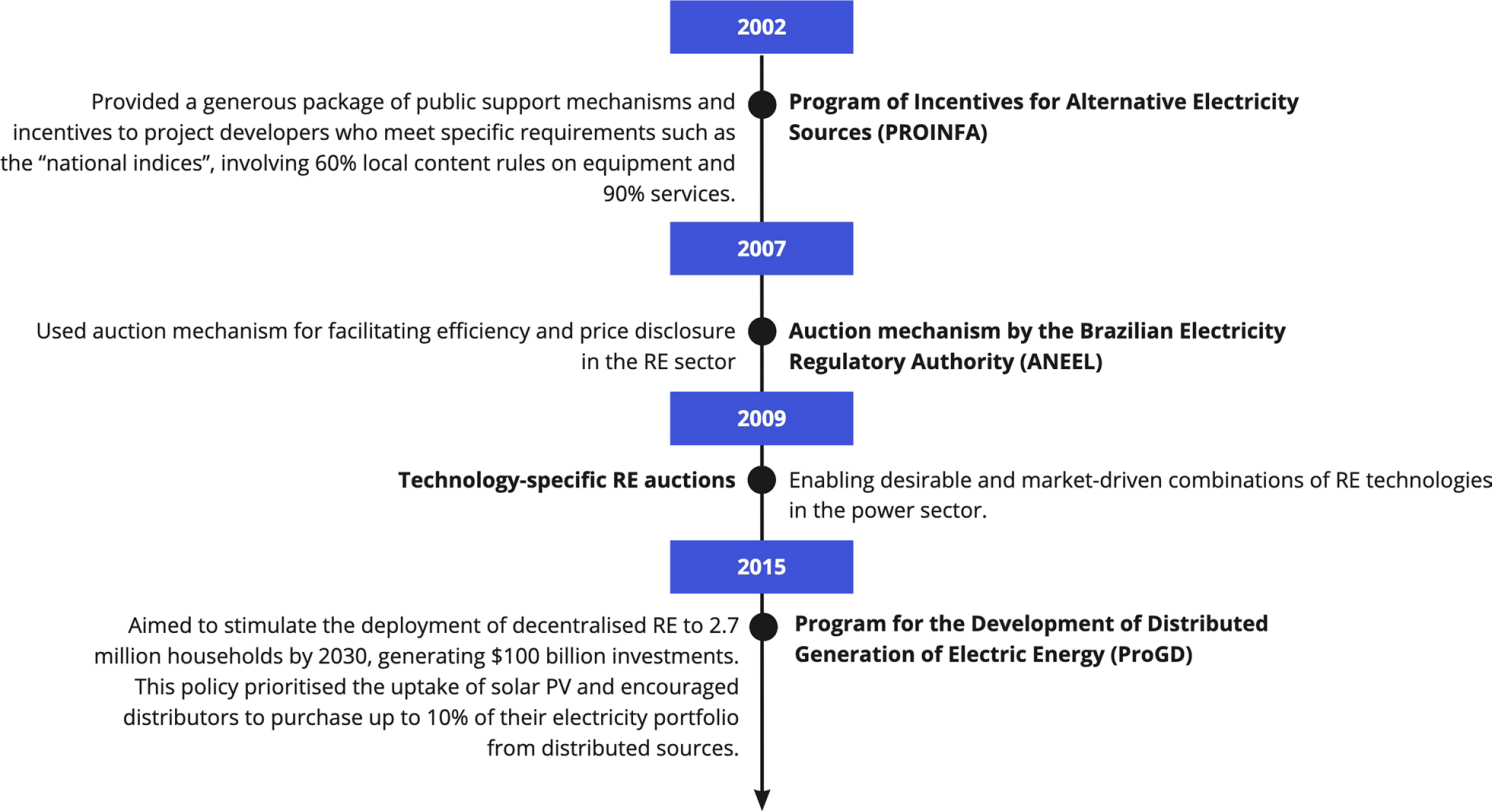
Key RE financing policy frameworks in Brazil

In Brazil, a significant policy push for wind and solar development began in 2002, following a drought that caused electricity shortages [44]. Figure 5 illustrates the key energy policies that have shaped the country’s financing environment for renewables. The Program of Incentives for Alternative Electricity Sources (PROINFA) was implemented to generate 3.3 GW of electricity from wind, biomass, and small hydro by 2006 [45]. It provided incentives to project developers who met the local content requirements [46]. This significantly boosted domestic capacity in wind turbine manufacturing, followed by a surge in the deployment of solar photovoltaics (PV) technology [17]. In addition, the policy mitigated off-taker and price risks through long-term contracts between developers and utilities. For example, the national utility, *Eletrobras*, was mandated to sign a 20-year power purchase agreement (PPAs) with RE project developers at attractive pre-determined tariffs. In addition, the program prioritized the procurement of energy from small independent power producers (IPPs) as a strategy to foster competition in the electricity sector.

The most important policy mechanism in Brazil was the introduction of auction mechanisms in 2007 to procure clean electricity. Project developers were to compete for RE contracts through frequent tenders which reduced costs and increased market innovation [47]. Most importantly for investors, auctions improved the financing environment by increasing transparency in electricity markets through clear price signals and fostering collaborations among developers, the national utility, and regulators (R4 and R12). Technology-specific policy frameworks to stimulate both the supply- and demand-sides of low-carbon technologies were another milestone. For example, technology-specific auctions helped to streamline the regulatory framework and priorities for procuring specific low-carbon technologies to meet rising electricity demand [48]. In addition, the Ministry of Mines and Energy (MME) introduced the Program for the Development of Distributed Generation of Electric Energy (ProGD) to stimulate the deployment of decentralized RE to 2.7 million households by 2030 and generate $100 billion in investments [49]. It stimulated demand by raising net metering for solar prosumers and requiring electricity distributors to purchase 10% of electricity from distributed renewable sources to access preferential funding from the Brazilian Development Bank (BNDES).

In sum, the confluence of supportive policy tools, periodic policy revisions, and positive feedback between public institutions and private stakeholders ensure a strong financing environment in Brazil.

### Financing channels for renewable energy

#### Public finance

Considering the significant investment risks facing novel low-carbon technologies in developing countries, public finance is essential to de-risk projects and attract private capital [7]. In Nigeria, government financing of RE projects is mostly channeled through fiscal allocations of federal and state governments. Recently, the federal government has tapped bond markets to raise capital for green projects. For example, it sold a sovereign green bond of $29 million to finance solar and forestry projects, with plans to issue an additional $150 billion by 2030 [50]. Administratively, public financing of decentralized clean energy has been led by the Rural Electrification Agency (REA), a sub-agency of the Ministry of Power that administers the Rural Electrification Fund (REF). The REA is funded through federal budget or loans from MDBs to build off-grid solar in remote communities, universities, and markets [51]. However, its lack of political independence, limited financial and investment management expertise, and focus on off-grid electrification in unserved communities have limited its capacity to mobilize the large amount of capital needed for utility-scale RE projects for a rapid energy transition.

On the other hand, public financing of renewables is more dynamic in Brazil. This is partly credited to the strong industrial policy levers that Brazilian policymakers have used to unlock financing for renewables [33]. For instance, both the PROINFA and Light for All policies were supported by two dedicated public financing mechanisms: The Global Reversion Reserve (RGR) and the Energy Development Fund (CDE). The RGR was funded through taxes and other fees imposed on electricity supply companies. Proceeds raised from these fiscal vehicles were channeled to financing off-grid projects under the Light for All program [52]. In addition, the CDE mobilized funds for PROINFA projects through a general increase in electricity tariffs for high-income consumers [44].

**Fig. 6:**
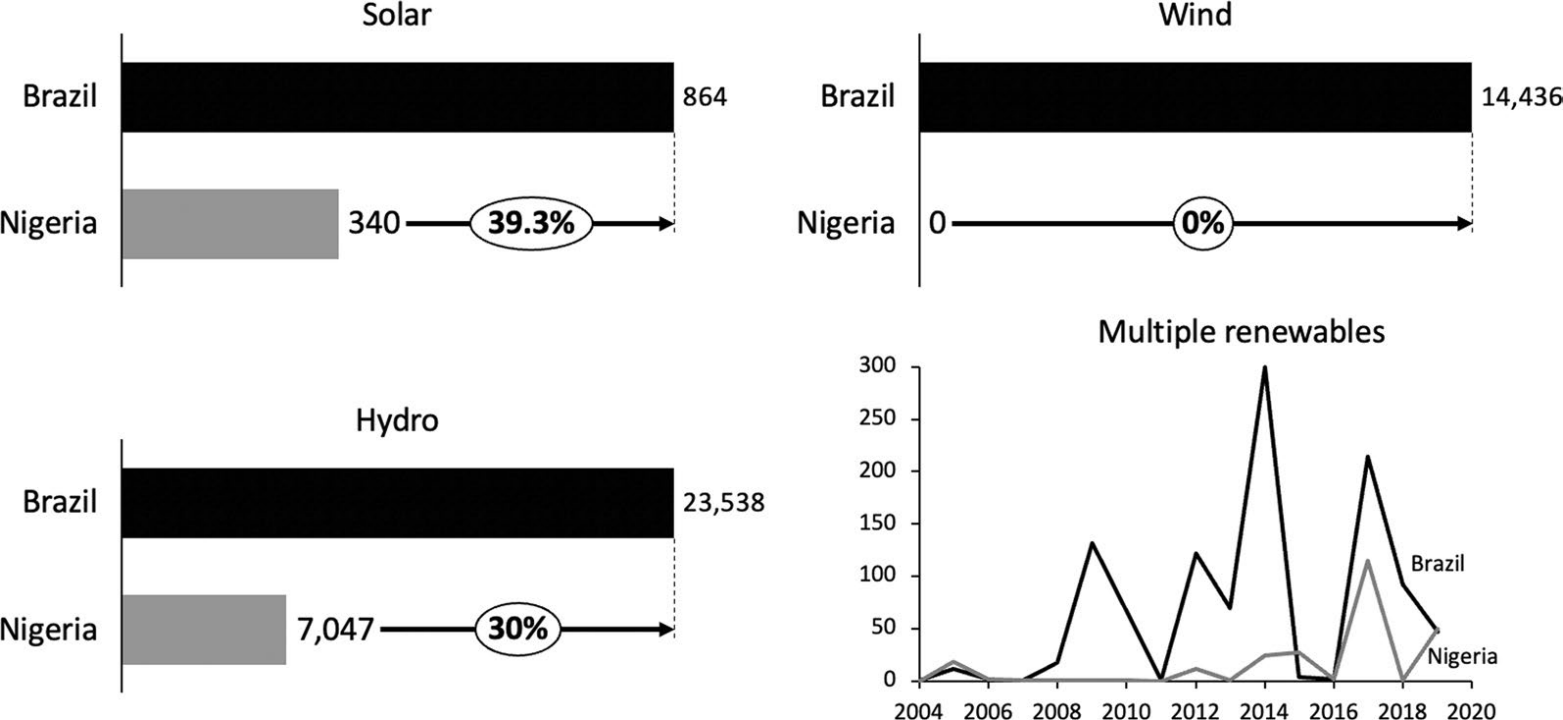
Flows of public finance into renewable energy in Brazil and Nigeria (2008–2019, US$ million)

Figure 6 situates the volume of public finance in the context of the current energy transition in Brazil and Nigeria. The International Renewable Energy Agency (IRENA) defines public finance as financial flows in the form of commitments originating from public institutions, such as governments, MDBs, and other public finance institutions. Public finance for renewables is significantly higher in Brazil than in Nigeria across solar, wind, and hydro technologies. Between 2008 and 2019, Brazil invested $864 million in solar, compared to $340 million in Nigeria. For wind technology, the gap is much wider, with public funding totaling $14.4 billion in Brazil relative to $5 million in Nigeria. The enormous scale of public investment in wind power in Brazil is largely due to a strong policy priority to wean the country off its dependence on hydropower and to develop domestic competitiveness in wind technologies [44]. In contrast, Nigeria had no substantive policy framework for harnessing wind resources [22]. Interestingly, total public investment in renewables was virtually similar in both countries in the early 2000s, but significantly diverged around 2007. This coincided with a period of increased policy stringency in Brazil, particularly with regard to revisions to PROINFA targets and the introduction of technology-specific auction mechanisms for renewables deployment [17, 48].

#### National development banks

Globally, state-owned development banks have been successful in financing RE projects and remain indispensable in the current energy transition [6, 7]. In Nigeria, the Bank of Industry (BoI) is gradually becoming a significant actor in financing renewables, with substantial potential to accelerate the energy transition. For example, its Access to Renewable Energy Program (AtRE) has so far extended $2 million in concessional debt financing for the construction of six solar mini-grids [53]. It recently reviewed its Solar Energy Fund into a 6 billion naira ($15 million) fund to finance distributed clean energy for micro-, small- and medium-scale enterprises [53]. However, major challenges for BoI include a lack of sufficient financial support from the Federal Government and weak collaborations with bilateral development agencies and MDBs (R5 and R7).

In contrast, BNDES has been the largest financier of RE in Brazil. It provides capital directly to project developers and indirectly through a partnership with public financial institutions such as Banco do Brasil and CAIXA, regional banks such as Banco Nordeste and Banco de Desenvolvimento de Minas Gerais, and private financial institutions [48, 54]. BNDES offers concessional financing for up to 80% of total project costs at 7–9% interest rates for up to 20 years. For instance, in 2002, it committed $2.3 billion to financing PROINFA renewables projects to boost private investments [44]. Between 2004 and 2018, BNDES accounted for over 70% of total debt financing to RE projects in Brazil [55]. It has also sold green bonds in international markets, using the proceeds used to finance sustainable energy [55].

However, the success of financing renewables in Brazil is not only due to the patient capital deployed by BNDES, but also to its dynamic role in forging public–private partnerships to mitigate risks for investors and foster learning in the financial industry. For example, it partners with domestic financial institutions (DFIs) to develop financing mechanisms to incentivize investment in green projects. The regional banks usually act as intermediaries between BNDES and project developers, thus helping to strengthen the capacity of local financial institutions to invest in novel technologies [17, 56]. Together with BNDES, DFIs also mobilize resources from other specialized public financing schemes, such as the Constitutional Funds created by the Ministry of National Integration [57]. In summary, BNDES’ strategic use of public finance to facilitate energy transition in Brazil is embedded in its mission-oriented approach to green industrial policy, which includes market-shaping, countercyclical, and stakeholder mobilization roles [58].

#### Bilateral agencies and multilateral development banks

**Fig. 7:**
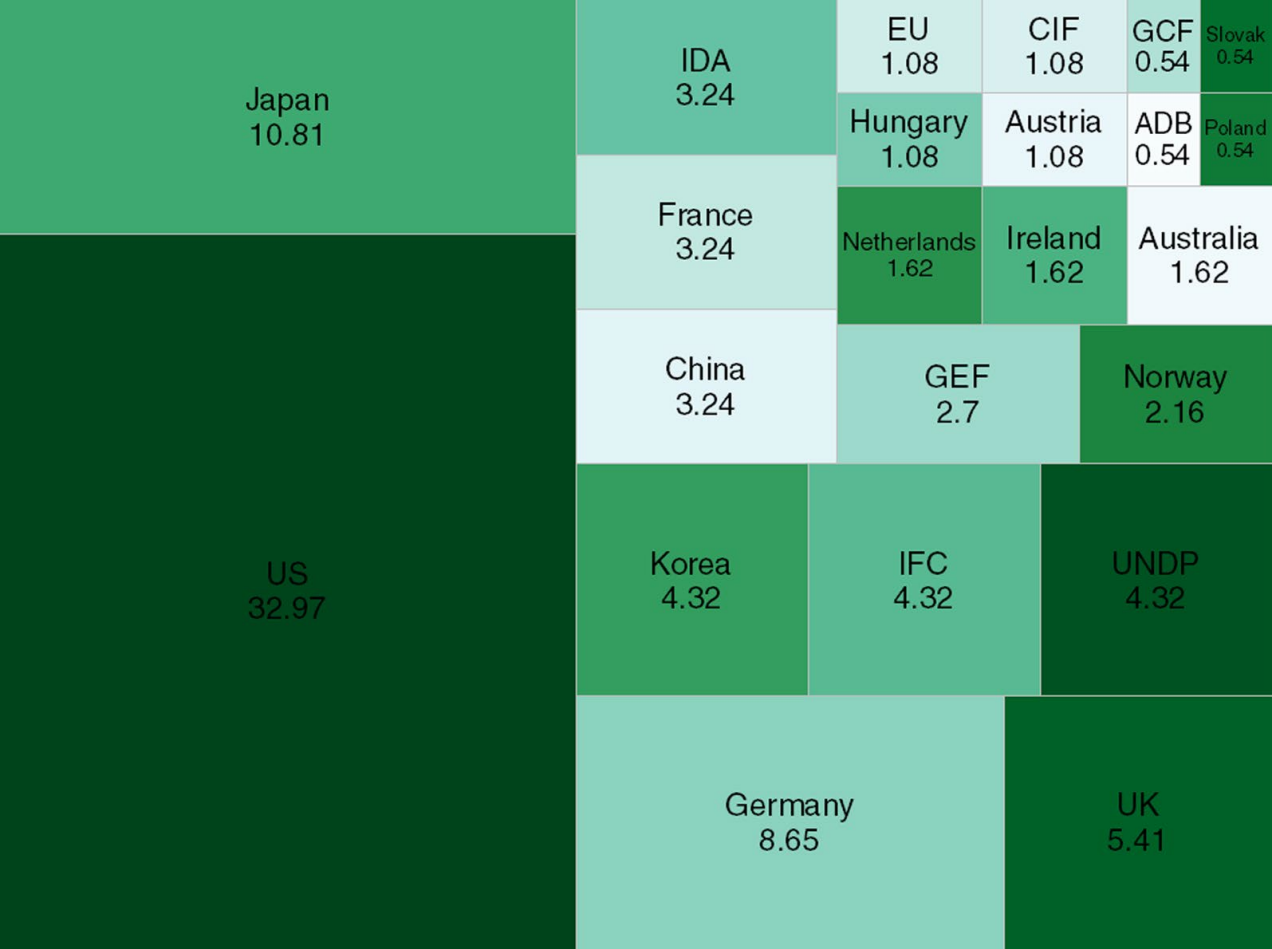
Shares of renewable energy finance from public sources in Nigeria (%)

The Nigerian RE sector is significantly dependent on development finance from development agencies and MDBs. This often consists of technical and financial support to policymakers, domestic financial institutions, and project developers. Edomah et al. [51] provide a comprehensive review RE interventions by various development organizations in Nigeria. Figure 7 shows that the U.S. (32.97%) is the largest provider of public finance for renewables in Nigeria, followed by Japan (10.81%), Germany (8.65%), and the UK (5.41%). These countries channel their RE interventions through their bilateral development agencies and private companies.

Based on research and expert interviews, we identify three fundamental challenges related to development finance of renewables in Nigeria. First, development agencies mostly operate in silos, financing small-scale and disaggregated RE projects. The lack of synergy and coordination among development stakeholders is counterproductive, considering the urgency of optimizing public finance to accelerate the energy transition. Second, donors often duplicate their efforts, leading to high transaction costs for both public and private stakeholders. For example, USAID’s Renewable Energy and Energy Efficiency Project (REEEP) and GIZ’s Nigeria Energy Support Program (NESP) have significant project overlap in terms of technical, advisory, and financial support in the RE sector [51]. Our interviewees associated this with increased transaction costs for developers and investors, leading to suboptimal outcomes in obtaining loans from domestic banks and attracting investment capital from foreign investors (R1, R4, and R6). According to our interviewees, large investors are mainly interested in bankable projects and are more likely to provide capital subject to significant public de-risking. However, such de-risking mechanisms, for example, via pooled public resources, could hardly be adequately achieved given the current political interests of donors (R1 and R4). Relatedly [59], note that without strong stakeholder coordination, RE investments would fall short of the scale needed to achieve energy access in African countries. In the “Discussion” section, we argue that an optimal strategy with higher potential multiplier effects would require donors to coordinate their financial resources and technical assistance to incentivize large-scale investments in renewable technologies.

**Fig. 8:**
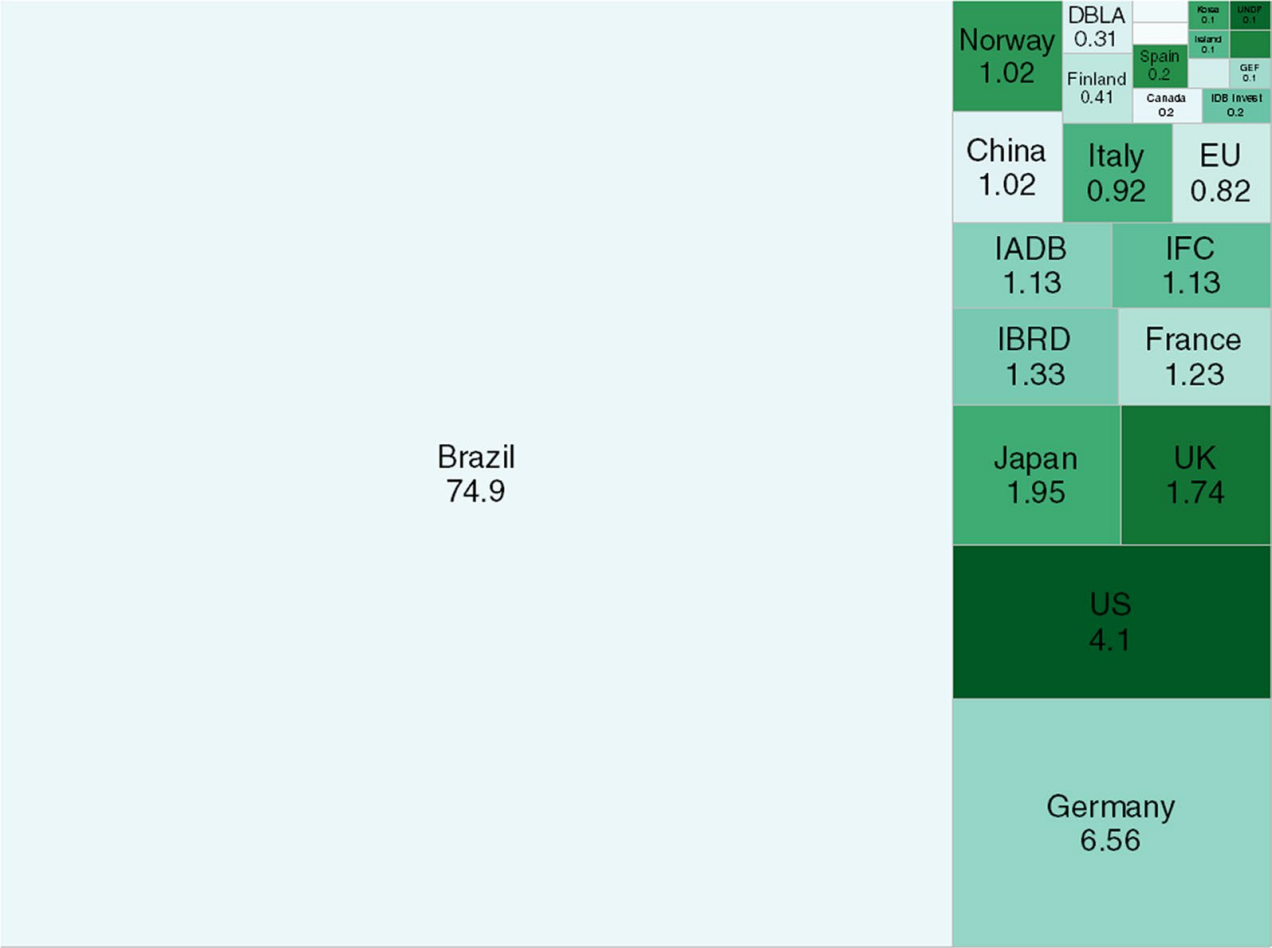
Shares of renewable energy finance from public sources in Brazil (%)

On the other hand, project developers in Brazil have not relied on funding from donors and MDBs. Figure 8 shows that the largest share of public finance for renewable energy comes from Brazil (74.9%), followed by Germany (6.56%), the U.S. (4.1%), and Japan (1.95%). The low share of foreign public finance could be partly attributable to a higher level of economic development and a relatively more liquid financial sector in Brazil. However, the substantial public financing provided by BNDES is arguably the most important factor in explaining the composition of public finance for renewables in the country. Indeed, BNDES invested over $78.8 billion in renewables between 2009 and 2018, while the combined investments of all MDBs and other development organizations amounted to only $1.3 billion [60].

#### Private financial institutions

In both countries, commercial banks have not played a significant role in financing RE projects. In Nigeria, for example, only a few Nigerian banks have provided financing for RE projects as of 2018. Possible reasons include technological risks of low-carbon technologies, high cost of capital (20–30%), scarcity of long-term credit, and banks’ lack of experience in managing RE portfolios (R7). However, some banks have begun financing renewables. In 2018, Ecobank financed mini-grids worth $600,000 backed by guarantees from USAID [61].

In Brazil, banks often participate with BNDES in syndicate loans. The availability of long-term, low-cost financing from BNDES creates incentives for banks to provide additional credit to developers with bankable projects [34, 54]. This is clear from R12’s comments that “*leading banks such as Banco Santander and Banco Bradesco have provided alternative debt and equity finance for RE developers who have won tenders and secured PPA with eletrobras*.” However, as the cost curves and project risks of wind and solar technologies have fallen substantially recently, banks are expected to play a larger role in the future. R12 asserted that “*recent policy changes regarding capital structure and interest rate by BNDES are expected to provide opportunities for increased participation from banks in the coming years.*”

In summary, Brazil’s rapid deployment of renewables, especially wind, has resulted from the strategic implementation of supportive policies, strong stakeholder coordination, and the deployment of public finance levers.

### Financing instruments for renewable energy

**Fig. 9:**
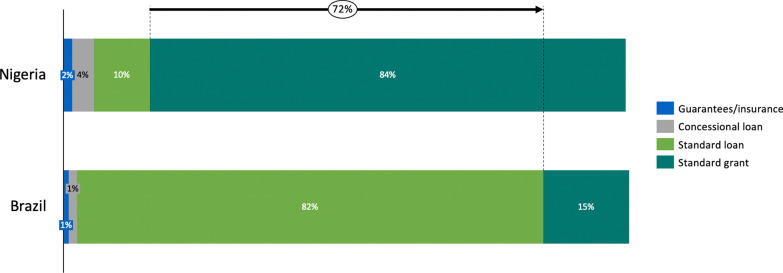
Share of financing instruments for renewables in Nigeria and Brazil

We have argued that different financing instruments are required to attract rapid investment in clean technologies in developing countries and emerging markets. Figure 9 shows the financing instruments for RE projects in Nigeria and Brazil. In Nigeria, standard grants from donors and MDBs account for 83.97% of public capital for project developers, followed by standard loans (9.92%), and concessional loans (3.82%). However, there are limited guarantees to mitigate systemic risks for investors. This is suboptimal considering the number of the foreign exchange, off-taker, technical, and policy risks faced by RE investors in Nigeria [38]. This is amplified in a comment by R8: “*Although several investors are interested in deploying capital in our project in northern Nigeria, they have expressed serious concerns around lack of attractive guarantees and consensus over put-call options agreements with the Federal government. With sufficient guarantees, the project will be bankable in the eyes of potential investors*”. Thus, diversifying financing instruments with a higher share of guarantees and concessional loans will significantly improve the risk-return profiles of RE projects in the country, thereby incentivizing private lending to renewables. Although BNDES has been the leading provider of loans to project developers in Brazil, private actors have also been important sources of green investment. For example, project developers and other non-financial firms account for 75% of total equity investment in the country. In addition, electricity stakeholders such as the state-controlled Eletrobras, State Grid Brazil, and Engie SA were prominent equity investors in onshore wind farms and small hydroelectric plants [17, 33].

On the other hand, private instruments are also pivotal in fostering the energy transition. For example, venture capitalists, angel investors, and private equity could provide early seed capital for RE start-ups in the death valley, helping them to scale [9]. In Nigeria, there is a shortage of early stage capital for RE entrepreneurs, making most start-ups to rely on the owner’s capital or debts and equities from informal sources (R1). However, there has been a steady increase in investments from foreign venture capital firms, particularly targeting mini-grid developers with innovative business models combining digital technologies and productive uses of energy [43].

## Discussion

The preceding sections identify key points of divergence and striking insights regarding the financing levers for RE investment and deployment in Nigeria and Brazil. Here, we synthesize the dominant themes of our analysis to propose policy implications for Nigeria. First, we underscore the need to ensure a sound financing environment in Nigeria, learning from the Brazilian experience. Second, we reflect on the mainstreaming of financing channels to leapfrog RE investment in Nigeria. Third, we argue that diversification of financing instruments can play a fundamental role in Nigeria’s evolving policy and fiscal regime(s) to incentivize RE investment.

### Creating a sound financing environment

Well-designed and credible policy tools, such as feed-in tariffs, auctions, and risk guarantees, are essential to creating an enabling financing environment for renewables [23, 33]. Brazil has leveraged these policy levers to facilitate its energy transition. In Nigeria, there has been limited political commitment to implementing policy mechanisms to promote private sector participation. A case in point is the PPA that the Nigerian government signed with 14 project developers in 2016 to develop 1.125 GW of solar electricity, which has been stalled due to policy reversals on feed-in tariffs and guarantees [38, 62]. This has increased sovereign risks and created uncertainty around the viability of large-scale RE projects in the country (R5). Thus, strong political will and policy coherence are needed to create a sound financing environment for the energy transition in Nigeria [22]. Other complementary policy levers include well-designed and technology-specific policy mechanisms, such as auctions, redirection of fuel subsidies towards renewables, and exchange rate stability through prudential monetary policy.

### Mainstreaming financing channels

**Fig. 10:**
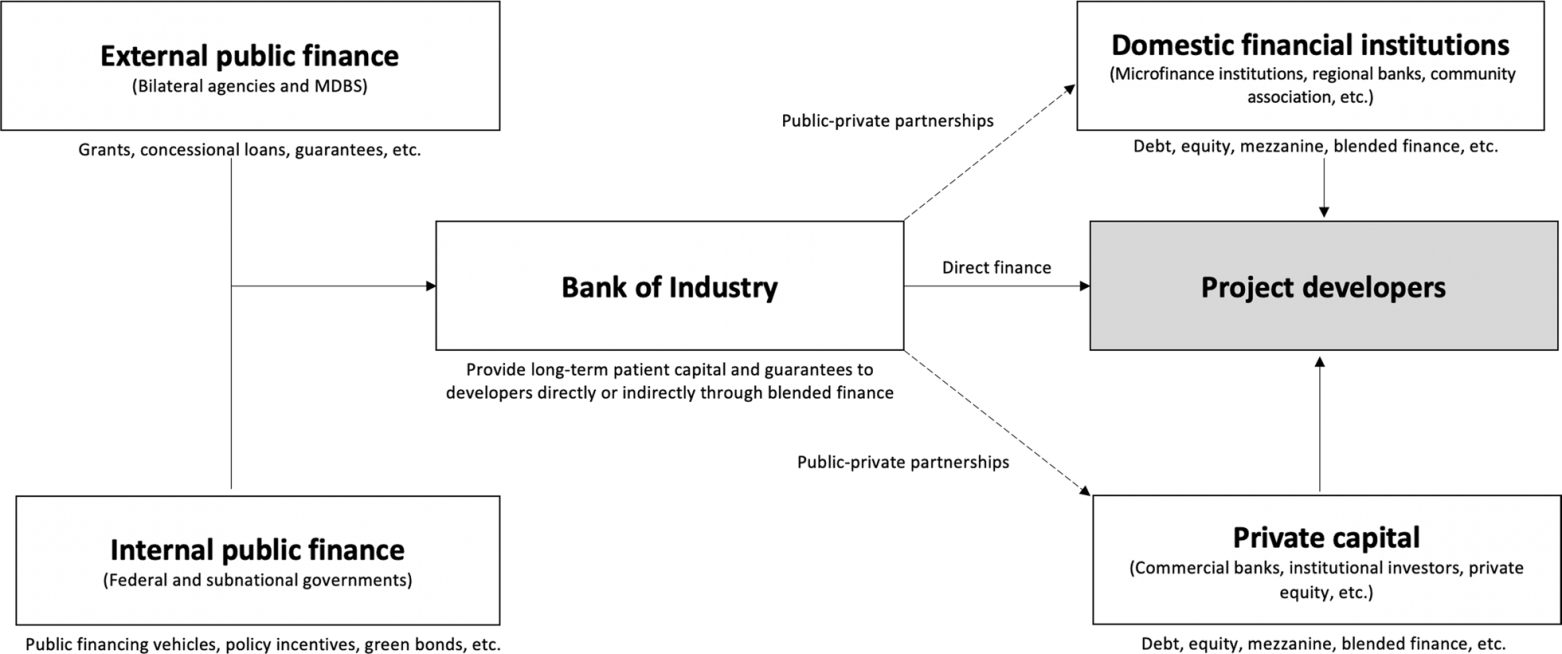
Proposed model for mainstreaming RE financing channels in Nigeria

Achieving energy transition in developing countries requires strong directionality from public financial institutions with extensive experience in financing dynamic local enterprises. In this regard, we propose that the Bank of Industry (BoI) should be reimagined to become a dynamic financier of RE in Nigeria. To strengthen its balance sheet, BoI can mobilize funds from public and private sources in domestic and international markets. For instance, it could tap into resources from institutional investors, such as pension funds and insurance companies. Similarly, it could borrow from special financing vehicles of the Central Bank of Nigeria (CBN) or the Ministry of Finance. Most importantly, development finance from MDBs and donors should be channeled through the BoI, which could blend it with resources from different stakeholders to provide long-term patient capital and guarantees to RE enterprises (see Fig. 10). This will enable the BoI to acquire strong balance sheets to finance and underwrite large-scale projects and attract more private capital.

It is believed that private actors often invest in renewables after public banks lead the way through financial learning [10]. Thus, the above model will unlock financing from Nigerian commercial banks for RE projects. Similar to the relationship between BNDES and other banks in Brazil, indirect financing from BoI could be provided through DFIs, such as microfinance banks through public–private partnerships, thereby accelerating learning in the financial industry while facilitating access to finance for RE enterprises. In addition, this would provide opportunities to achieve a stronger representation of community-based organizations (CBOs) in financing RE projects, thereby facilitating financial inclusion and a just energy transition [43].

### Diversifying financing instruments

Supporting the energy transition in developing countries requires a diverse portfolio of financial instruments specifically tailored to overcome the risks faced by developers. Particularly, policymakers in Nigeria should select instruments that most effectively leverage private investment [63]. Potential instruments include blended finance, mezzanine securities, and risk guarantees, which the BoI could take the lead in structuring alongside other financial institutions. However, policymakers also need to be aware of the pros and cons of these alternative financial structures, as well as their synergies and tradeoffs, especially as technologies mature overtime. At the macro level, financial regulators could encourage the reallocation of capital to clean energy by mandating climate disclosures and targets for financial institutions in Nigeria. More broadly, clear policy signals and diverse financial structures will help cater to different types of investors with heterogeneous risk sentiments. It will also ensure flexibility for developers to select the instruments that best fit their business models and risk-return profiles.

## Conclusions

Achieving climate targets will require a global transition to clean energy. However, financial, regulatory, and economic barriers impede investment in low-carbon technologies. This paper provided a comprehensive analysis of energy transition landscapes in Nigeria and Brazil by analyzing RE financing environments, channels, and instruments.

We found that while Brazil has achieved remarkable success in galvanizing financing for renewable technologies, Nigeria has failed to achieve similar success due to several challenges, including high policy risks, insufficient political will, limited financing instruments, and weak public-private partnerships among financial stakeholders. While RE investment in Brazil has been driven by public financing from BNDES, bilateral agencies and MDBs have been the largest financiers of RE in Nigeria. Our research showed that lack of strong coordination among bilateral agencies and MDBs in Nigeria has inadvertently increased transaction costs and risk profiles of renewable technologies. We developed a new multi-stakeholder financing framework to consolidate public and private capital to accelerate the mobilization of investment for energy transition in Nigeria.

We made three policy recommendations. First, Nigerian policymakers should ensure a sound financing environment with favorable regulations, credible policy implementation, and technology-specific support mechanisms for renewables. Second, policymakers should mainstream financing channels by promoting strong partnerships among public and private financial stakeholders, spearheaded by the BoI, to mitigate risks for investors and developers. Third, there is a need to diversify financing instruments to account for commercial and political risk appetite of different investor types. In particular, more guarantee instruments that facilitate risk-sharing will help to unlock additional private capital for renewables.

While this study has focused on Nigeria and Brazil, future research could cover more LMICs to provide a broader perspective. In addition, further research using rigorous quantitative methods, for example, policy and investment modeling, could add novelty to the literature. Finally, conceptual and empirical studies that combine rigorous analysis of how systemic financial levers affect project-level risk-return profiles of RE technologies could be an interesting improvement.

## Data Availability

All data used are publicly available and carefully referenced in the paper.

## Abbreviations

AfDB: African Development Bank
ANEEL: Brazilian Electricity Regulatory Agency
AtRE: Access to RE Programme
BloombergNEF: Bloomberg New Energy Finance
BNDES: Brazilian Development Bank
CDE: Energy Development Fund
COP: Conference of the Parties
CTF: Clean Technology Fund
DisCos: Distribution Companies
ECN: Energy Commission of Nigeria
EPE: Energy Research Office
ESP: Economic Sustainability Plan
FCDO: Foreign, Commonwealth and Development Office
FiT: Feed-in tariff
GenCos: Generation Companies
GIZ: German Development Agency (GIZ)
GOGLA: Global Off-grid Lighting Association
GW: Gigawatts
IDCOL: Infrastructure Development Company Limited
IEA: International Energy Agency
IPP: Independent power producers
IRENA: International Renewable Energy Agency
LMICs: Low- and middle-income countries
MDBs: Multilateral development banks
MFI: Microfinance institutions
MME: Ministry of Mines and Energy
MoE: Ministry of Environment
MoP: Ministry of Power
MW: Megawatts
MYTO: Multi-year tariff order
NBET: Nigeria Bulk Electricity Trading
NEEAP: National Energy Efficiency Action Plan
NEP: Nigeria Electrification Project
NEPA: National Electric Power Authority
NERC: Nigerian Electricity Regulatory Commission
NESP: Nigerian Energy Support Program
NREAP: National Renewable Energy Action Plan
NREEEP: National Renewable Energy and Energy Efficiency Policy
PPA: Power purchase agreement
ProGD: Program for the Development of Distributed Generation of Electric Energy
PROINFA: Program of Incentives for Alternative Electricity Sources
RE: Renewable energy
REA: Rural Electrification Agency
REAN: Renewable Energy Association of Nigeria
REEEP: Renewable Energy and Energy Efficiency Project
REF: Rural Electrification Fund
REG: Global Reversion Reserve
REP: Renewable Energy Programme
RMP: Renewable Energy Masterplan
SE4All: Sustainable Energy for All
SHS: Solar home systems
TWh: Terra-watt hours
UNEP: United Nations Environment Programme
USADF: US African Development Foundation
USAID: United States Agency for International Development
